# Methylated *SFRP2* and* SDC2* in stool specimens for Colorectal Cancer early detection: A cost-effective strategy for Chinese population

**DOI:** 10.7150/jca.52478

**Published:** 2021-03-05

**Authors:** Guodong Zhao, Xiaoyu Liu, Yi Liu, Yong Ma, Jun Yang, Hui Li, Shangmin Xiong, Sujuan Fei, Minxue Zheng, Xiangwei Zhao

**Affiliations:** 1 State Key Laboratory of Bioelectronics, School of Biological Science and Medical Engineering, Southeast University, Nanjing 210009, China.; 2 Zhejiang University Kunshan Biotechnology Laboratory, Zhejiang University Kunshan Innovation Institute, Kunshan Jiangsu 215300, China.; 3 Suzhou Institute of Biomedical Engineering and Technology, Chinese Academy of Sciences, Suzhou Jiangsu 215163, China.; 4 Department of Gastroenterology, Affiliated Hospital of Xuzhou Medical University, Xuzhou Jiangsu 221002, China.; 5 Suzhou VersaBio Technologies Co. Ltd., Kunshan Jiangsu 215300, China.; 6 Institute of Digestive Diseases, Xuzhou Medical University, Xuzhou Jiangsu 221002, China.

**Keywords:** colorectal cancer, stool, m*SFRP2*, * mSDC2*, early detection

## Abstract

**Background:** The aim of this study was to evaluate the feasibility of combination of methylated *SFRP2* and methylated *SDC2* (SpecColon test) in stool specimens for colorectal cancer (CRC) early detection and to optimize the cut-off value of methylated *SFRP2* and methylated *SDC2*.

**Methods:** Approximately 5 g of stool specimen each was collected from 420 subjects (291 in the training cohort and 129 in the validation cohort). Stool DNA was extracted and bisulfite-converted, followed by detection of methylated level of *SFRP2* and* SDC2*. Youden index was employed to determine the cut-off value.

**Results:** The whole operating time for stool SpecColon test takes less than 5 hours. The limit of detection of combination of methylated *SFRP2* and methylated *SDC2* was as low as 5 pg per reaction. The optimized cut-off value was methylated *SFRP2* analyzed by 3/3 rule and methylated *SDC2* analyzed by 2/3 rule. In the training cohort, the sensitivities of stool SpecColon test for detecting AA and early stage CRC (stage 0-II) were 53.8% (95% CI: 26.1%-79.6%) and 89.1% (95% CI: 77.1%-95.5%) with a specificity of 93.5% (95% CI: 87.2%-96.9%), and the AUC for CRC diagnosis was 0.879 (95% CI: 0.830-0.928). Similar performance was achieved by SpecColon test also in the validation cohort, where its sensitivities for detecting AA and early stage CRC (stage 0-II) were 61.5% (95% CI: 32.3-84.9%) and 88.5% (95% CI: 68.5%-97.0%) with a specificity of 89.5% (95% CI: 74.3-96.7%).

**Conclusion:** Combined detections of methylated *SFRP2* and methylated *SDC2* in stool samples demonstrated high sensitivities and specificity for the detection of AA and early stage CRC. Therefore, this combination has the potential to become an accurate and cost-effective tool for CRC early detection.

## Introduction

As one of the most common malignant tumors, colorectal cancer (CRC) accounted for approximately 2 million new cases and over 881,000 deaths worldwide in 2018 1, including over 521,490 new CRC cases and more than 303,853 deaths for the same year in China 2. In order to reduce the incidence and mortality rates of CRC, many strategies have been employed for CRC early detection, such as guaiac-based fecal occult blood test (gFOBT) and colonoscopy 3. A long-term follow-up study in UK revealed the adenoma removal after CRC screening and the following regular colonoscopy surveillance could reduce the CRC risk among 2/3 patients 4. The CRC screening program follow-up results in USA also indicated the CRC incidence and mortality rates have declined significantly since the colonoscopy was effectively implemented 1. However, colonoscopy screening for CRC and adenoma is expensive and not sufficiently available in many resource-limited rural areas in China. Meanwhile, due to its invasiveness and bothersome bowel preparation, colonoscopy only elicited a relatively low participation and compliance rate in China 5, 6. Given the large population and an underdeveloped economy, the effective CRC early detection method must be accurate, safe, cost-effective and easy to operation. At-home cancer screening with new technologies allowing self-sampling by patients themselves at home can be a cost-effective solution for China and other developing countries with large populations to improve early detection rate 7.

Abnormal DNA methylation has been demonstrated in many cancers 8. A number of methylation biomarkers have been found to be associated with CRC and precancerous lesions in stool or plasma samples 9, 10. In recent years, several non-invasive methods based on blood or stool methylation biomarkers have been developed for CRC early detection 11-14. We had previously demonstrated high sensitivity and specificity of a plasma-based multiplex methylated DNA test, SpecColon, for CRC early detection 15. It detects methylated *SFRP2* (m*SFRP2)*, methylated *SDC2* (m*SDC2*) and an internal control gene (*ACTB*) simultaneously in a single qPCR reaction. The test detected 58.3% advanced adenomas (AA) and 76.2% CRC with 1 mL plasma, demonstrating its potential as an accurate and cost-effective strategy for CRC early detection. In comparison, one of the other methylation biomarkers for CRC early detection, methylated *SEPT9*, detected AA and stage I CRC with significantly lower sensitivities, 10-20% and less than 50% respectively, with plasma samples 16, 17. In this study, we evaluated the feasibility of SpecColon test for stool specimens as an at-home screening method for CRC early detection and optimized its cut-off value. Therefore, SpecColon test that can be applied with either blood or stool could facilitate broader population to participate in the early diagnosis of colorectal cancer.

## Materials and Methods

### Sample collection

**Figure 1:**
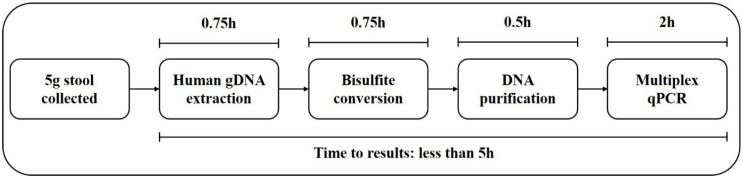
Outline of the stool SpecColon test work flow.

From July 1, 2018 to January 10, 2020, a training cohort including 309 stool specimens with colonoscopy results were collected from the Affiliated Hospital of Xuzhou Medical University. From March 1, 2020 to November 30, 2020, an independent validation cohort including 131 stool specimens were collected from the same hospital. The inclusion criteria consisted of the following: aged 18 or older, no history of CRC, no pregnant woman, having colonoscopy results, and participants with abnormal colonoscopy results should have pathological diagnosis results. During stool sample collection, efforts were made to avoid transferring urine into the collection tube, and diarrhea samples were not collected. The exclusion criteria of stool samples were as follows: missing or incomplete sample information, insufficient or excessive stool volume, repeated sampling, and insufficient DNA indicated by low *ACTB* levels (see data analysis). After exclusion of invalid samples, 291 subjects in the training cohort (111 CRC patients, 13 AA patients 12, 44 patients with small polyps (SP) 15 and 123 healthy subjects with no evidence diseases (normal subjects)) and 129 subjects in the validation cohort (58 CRC patients, 13 AA patients, 20 SP patients and 38 normal subjects) were included for final analysis. The diagnoses of the patients were histologically confirmed by the pathologist (Table 1). Stools were collected prior to bowel preparation or colonoscopy in single-use disposable sampling boxes (Suzhou VersaBio Technologies Co. Ltd., Kunshan, Jiangsu, China) mounted on toilet seats, and approximately 5 g of each fecal specimen was transferred into a 50 mL centrifuge tube containing 25 mL of preservative buffer (Suzhou VersaBio Technologies Co. Ltd.) to stabilize human genomic DNA in the stool (Figure 1)^18^. All stool samples were stored at room temperature for no more than 7 days and transferred to -80 °C for long term storage before usage. All participants have signed the informed consent and this study was approved by the Institutional Review Board of the Affiliated Hospital of Xuzhou Medical University (XYFY2018-KL081).

### DNA extraction, bisulfite treatment and quantitative real-time PCR

The stool SpecColon test work flow is outlined in Figure 1. The human genomic DNA in stool specimens was extracted with a stool DNA extraction kit (Suzhou VersaBio Technologies Co. Ltd.) following the manufacturers' instructions. Firstly, after 1 min homogenization, each fecal sample was centrifuged for 20 min at 10,000 g to remove particulate matter, and 500 μL preservative buffer was added into 0.15 mL supernatant before centrifuged at 20,000 g for 3 min. The resulting supernatant was then transferred to a new tube, 600 μL lysis buffer and 20 μL proteinase K were added into each supernatant followed by incubation at 70 °C for 10 min. Next, each supernatant was added with 600 μL ethanol and then loaded onto a spin column. The DNA was eluted with 100 μL elution buffer after washing for twice. Using a fast bisulfite conversion kit (Suzhou VersaBio Technologies Co. Ltd.), eluted DNA was converted and purified by adding 150 μL conversion buffer and 25 μL protection buffer, followed by incubation at 80 °C for 45 min. Finally, two washing steps were performed, and the column was air dried and DNA was eluted with 100 μL elution buffer 18.

Purified DNA obtained from the above steps was tested with SpecColon test in three PCR replicates (Suzhou VersaBio Technologies Co. Ltd.) 15. The qPCR reaction volume was 30 μL with 15 μL PCR mastermix and 15 μL DNA. qPCR was performed on LC480-II thermal cycler (Roche Diagnostics) according to a previous publication 15: activation at 95 °C for 30 minutes, 50 cycles of 95 °C for 10 seconds, 58 °C for 30 seconds, 72 °C for 10 seconds, and final cooling to 40°C for 30 seconds.

### SpecColon performance analysis

To determine the limit of detection (LoD) of SpecColon test for detecting m*SFRP2* and m*SDC2* DNA, different amounts of fully-methylated genomic DNA (2, 4, 6, 8, 10, 15, 20 pg/qPCR reaction) were diluted into unmethylated genomic DNA to create mixtures 12. SpecColon test was performed in 24 replicates at each concentration to determine LoD, which is defined as the lowest target concentration that produces positive result in more than 95% of replicate experiments 19. Each test result was considered 'detected' if *ACTB* Cp was less than 40.0 and the Cp values of methylated m*SFRP2* and m*SDC2* were less than 45.0 and 50.0, respectively.

### Data analysis

A stool sample was excluded if Cp value of* ACTB* was greater than 40.0, and m*SFRP2* and m*SDC2* were regard as 'detected' if their Cp values were less than 40.0 and 50.0, respectively. As a multiplex qPCR reaction run in triplicates, SpecColon test could return several possible results depending on different cut-off values. According to this principle, the results of SpecColon test were analyzed with two different cut-off values (Tables 1 and 2). For cut-off-1, both m*SFRP2* and m*SDC2* were analyzed by 2/3 rule in which a sample was scored positive if either m*SFRP2* or m*SDC2* was 'detected' in two of three replicates with valid amplification curves. For cut-off-2, m*SFRP2* was analyzed by 3/3 rule, while m*SDC2* was analyzed by 2/3 rule. A stool sample would be called positive by SpecColon test if either m*SFRP2* or m*SDC2* was positive. Youden index (i.e., sensitivity + specificity - 1) was applied to determine optimal cutoff values, where sensitivity is the positive detection rate of AA or CRC, and specificity is 1 minus positive detection rate of control. Therefore, Youden index of AA or CRC equals to positive detection rate of AA or CRC minus that of control. The methylated levels of m*SFRP2* or m*SDC2* in stool samples were analyzed using their mean Cp values.

Statistical analysis was performed using IBM SPSS for Windows, Version 22.0, and *t*-test was used for the comparison between two testing subjects at the significance level of *p*<0.05. Receiver operating characteristic (ROC) curves were plotted also using IBM SPSS.

## Results

Two hundred and ninety-one participants in total were included in this study as the training cohort for optimizing the cutoff values of stool SpecColon test, and the baseline characteristics of the subjects are shown in Table 1. Among all subjects, 123 were from normal healthy subjects, 111 from CRC patients (including 3 stage 0, 20 stage I, 32 stage II, 37 stage III, 10 stage IV patients and 9 patients of unknown stage), 57 from polyp patients (including 13 AA patients and 44 SP patients). The ages of all CRC patients ranged from 27 to 84 with a mean age of 60.8, and 56.8% were male patients. The ages of normal subjects ranged from 23 to 83 with a mean age of 46.9, and 53.7% were male subjects (Table 1).

**Figure 2:**
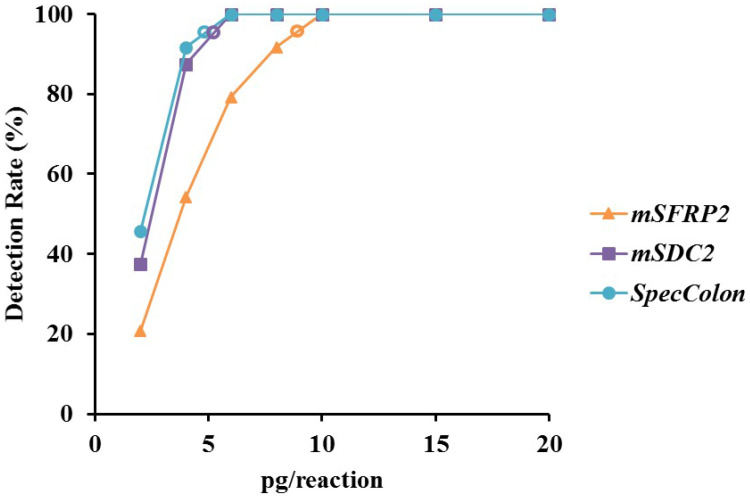
LoD (hollow circle) analysis of m*SFRP2* (orange), m*SDC2* (purple) and SpecColon test (blue) by using DNA solutions of different methylation levels.

To evaluate the analytical performance of m*SFRP2* alone, m*SDC2* alone and SpecColon test, mixtures of different ratios of bisulfite-treated fully-methylated and unmethylated genomic DNA were each tested for 24 replicates. As shown in Figure 2, both m*SFRP2* and m*SDC2* could detect 2 pg/reaction methylated genomic DNA with detection rates of 20.8% and 37.5%, respectively, while for the combination of mSFRP2 and m*SDC2*, the positive detection rate of SpecColon test in 2 pg/reaction increased to 45.8%. The LoD of m*SFRP2* alone, m*SDC2* alone and SpecColon test were approximately 9 pg, 6 pg and 5 pg, respectively, indicating that the combination of m*SFRP2* and m*SDC2* could achieve higher sensitivity than either single biomarker.

In order to evaluate the performance of stool SpecColon test more accurately, we employed two cut-off values to analyze the samples from AA, CRC, SP patients and normal individuals. The main difference was that m*SFRP2* was analyzed by 2/3 rule for cut-off-1 and 3/3 rule for cut-off-2. For cut-off-1, m*SFRP2* was detected in 58.8% (7/13) of AA, 66.7% (2/3) of stage 0, 85.0% (17/20) of stage I, 87.5% (28/32) of stage II, 81.1% (30/37) of stage III, 90.0% (9/10) of stage IV, and 88.9% (8/9) of unknown stage CRC samples (Table 1). The sensitivities for detecting AA and CRC by m*SFRP2* alone were 53.8% (95% CI: 26.1-79.6%) and 84.7% (95% CI: 76.3-90.6%), respectively, with a specificity of 87.0% (95% CI: 79.4-92.2%) (Table 2). For cut-off-2, m*SFRP2* was detected in 30.8% (4/13) of AA, 66.7% (2/3) of stage 0, 70.0% (14/20) of stage I, 84.4% (27/32) of stage II, 64.9% (24/37) of stage III, 50.0% (5/10) of stage IV, and 77.8% (7/9) of unknown stage CRC samples (Table 1). The sensitivities for detecting AA and CRC by m*SFRP2* alone decreased to 30.7% (95% CI: 10.3-61.1%) and 71.2% (95% CI: 61.7-79.2%), respectively, but the specificity was improved to 94.3% (95% CI: 88.2-97.5%) (Table 2). m*SDC2* was detected in 46.2% (6/13) of AA, 66.7% (2/3) of stage 0, 75.0% (15/20) of stage I, 81.3% (26/32) of stage II, 75.7% (28/37) of stage III, 60.0% (6/10) of stage IV, and 66.7% (6/9) of unknown stage CRC samples for both cut-off-1 and cut-off-2 (Table 1). The sensitivities for detecting AA and CRC by m*SDC2* alone were 46.2% (95% CI: 20.4-73.9%) and 74.8% (95% CI: 65.5-82.3%), respectively, with a specificity of 98.4% (95% CI: 93.7-99.7%) (Table 2).

However, when m*SFRP2* and m*SDC2* were combined for cut-off-1 or cut-off-2, the sensitivities of stool SpecColon test for detecting AA and CRC both showed improvement. The sensitivities of SpecColon test by cut-off-1 for AA and CRC were 61.5% (95% CI: 32.3-84.9%) and 89.2% (95% CI: 81.5-94.0%), respectively, with a specificity of 87.0% (95% CI: 79.4-92.2%) (Table 2). And the sensitivities of SpecColon test by cut-off-2 for AA and CRC were 53.8% (95% CI: 26.1-79.6%) and 83.8% (95% CI: 81.5-94.0%), respectively, with a specificity of 93.5% (95% CI: 87.2-96.9%) (Table 2). Therefore, cut-off-1 achieved relatively higher sensitivities but a lower specificity, while cut-off-2 showed relatively lower sensitivities with a higher specificity. To choose the optimal cut-off value, Youden indices of SpecColon test for cut-off-1 and cut-off-2 were calculated, showing that a higher Youden index was achieved by cut-off-2 for both AA and CRC detection (Table 2). Therefore, all subsequent data were analyzed by cut-off-2.

**Figure 3:**
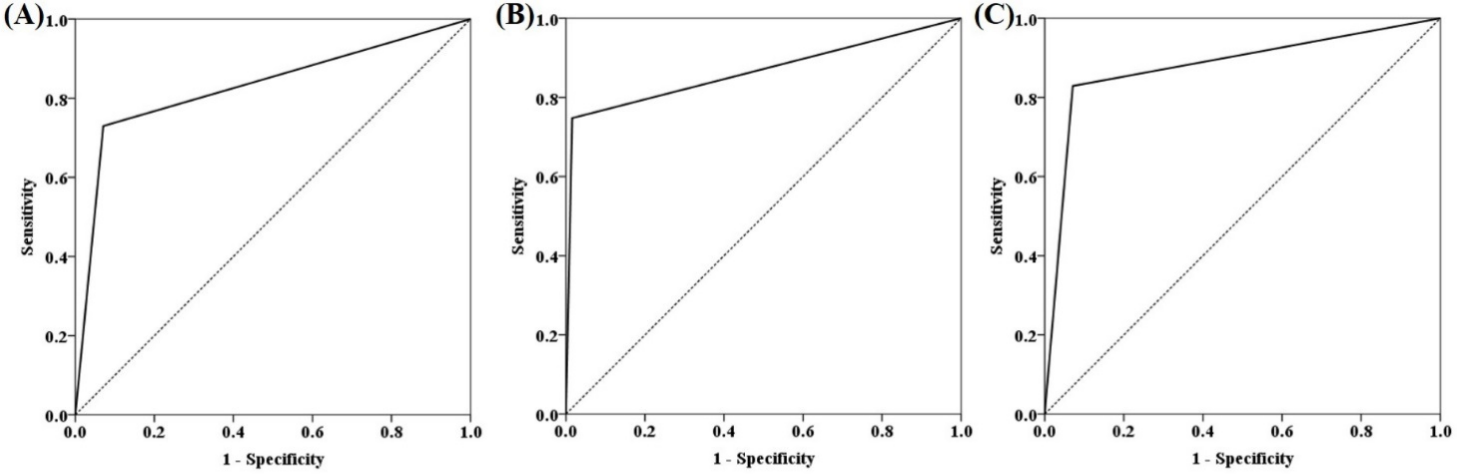
ROC curves for m*SFRP2* (A), m*SDC2* (B) and SpecColon test (C) in detecting CRC in the training cohort.

**Figure 4:**
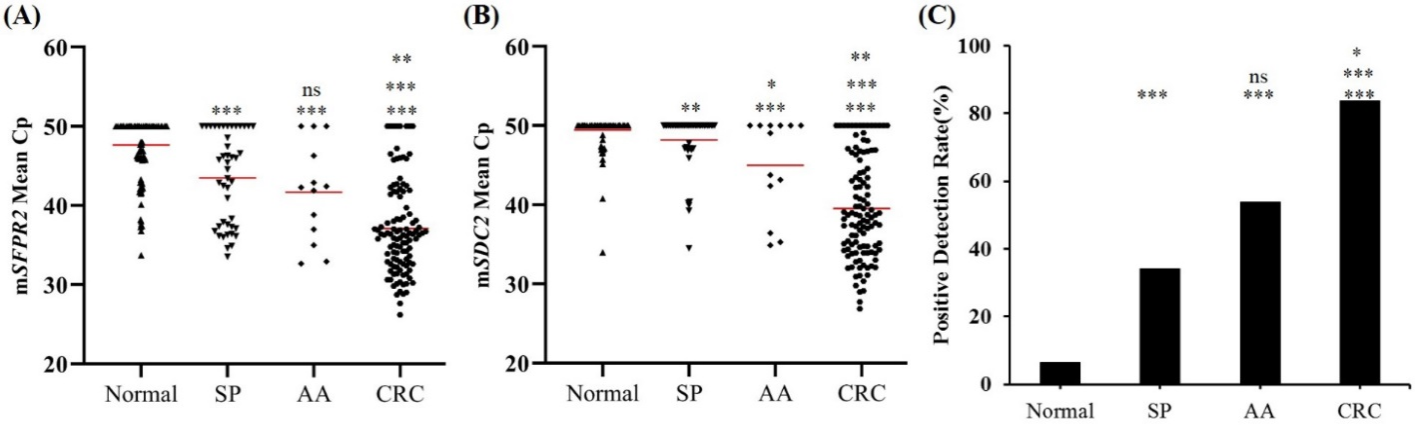
SpecColon stool test analysis in stool samples from training cohort for normal individuals, SP, AA and CRC. (A) Methylated levels (mean Cp values) of *SFRP2* gene; (B) Methylated levels (mean Cp values) of *SDC2* gene; (C) Positive detection rates of SpecColon test in the training cohort. Horizontal red bars denote the mean, ns not significant, *p < 0.05, **p < 0.01, ***p < 0.001.

ROC curves for m*SFRP2*, m*SDC2* and SpecColon test for CRC detection in the training cohort are shown in Figure 3. AUC for m*SFRP2* alone and m*SDC2* alone were 0.829 (95% CI: 0.773-0.886) and 0.866 (95% CI: 0.815-0.917), respectively. In contrast, SpecColon test improved AUC to 0.879 (95% CI: 0.830-0.928). The results of DNA methylated levels showed the mean Cp values of *SFRP2* and *SDC2* in normal individuals were both significantly higher than those in SP, AA and CRC patient groups (Figure 4A and B). And the mean Cp values of m*SFRP2* and m*SDC2* in CRC groups were also significantly higher than those in SP groups (*p*<0.0001). Furthermore, the positive detection rates for SP group and AA group showed no significant difference (*p*=0.199), whereas CRC group had significantly higher positive detection rate than normal control group and SP group (*p*<0.001, Figure 4C), and AA group showed significantly higher positive detection rate than normal control group (*p*<0.001, Figure 4C). For different characteristics analysis, although the positive detection rates of m*SDC2* alone showed differences among the different genders and tumor locations (*p*<0.05, Table 3), but there were no significant differences for SpecColon test among different ages, genders, stage, tumor locations, and tumor sizes (*p*>0.05, Table 3).

To validate the performance of stool SpecColon test, another independent validation cohort including 58 CRC, 13 AA, 20 SP patients and 38 normal control subject were recruited (Table 4). The results revealed that the sensitivities of m*SFPR2* alone for detecting AA and CRC were 61.5% (95% CI: 32.3-84.9%) and 77.6% (95% CI: 64.4-84.1%), the sensitivities of m*SDC2* alone for detecting AA and CRC were 46.2% (95% CI: 20.4-73.9%) and 86.2% (95% CI: 74.1-93.4%). When m*SFRP2* and m*SDC2* were combined in SpecColon test, the sensitivities for detecting AA and CRC were improved to 61.5% (95% CI: 32.3-84.9%) and 89.7% (95% CI: 78.2-95.7%). The specificities of m*SFPR2* alone, m*SDC2* alone and SpecColon test were 89.5% (95% CI: 74.3-96.7%), 97.4% (95% CI: 84.6-99.9%) and 89.5% (95% CI: 74.3-96.7%), respectively.

## Discussion

Early detection is the key strategy for reducing the incidence and mortality rates of CRC. Several developed countries have established long term CRC screening programs since 2000 and achieved significant reduction of CRC incidence in the past decade 1. However, in developing countries such as China, due to large populations and limitations of medical staff and infrastructure, current screening strategies have suffered from low participation and compliance rates 5. Thus, cost-effective CRC early detection methods with simple operation steps and flexible application scenarios are more suitable in China. Recently, our research team described a novel methylation DNA test, SpecColon, that could detect 58.3% AA and 76.2% stage I-IV CRC with a specificity of 87.9% in plasma samples 15, thus providing a lower cost, convenient and highly effective early detection tool for CRC screening in China. In this study, we optimized the cut-off value of SpecColon test in stool specimens and evaluated the feasibility of stool SpecColon test for CRC early detection.

The results showed that the sensitivities of stool SpecColon test for detecting AA, early stage (0-II) CRC and stage I-IV CRC by cut-off-2 were 53.8% (7/13), 89.1% (49/55) and 83.8% (93/111), respectively, with a specificity of 93.5%. Compared with plasma SpecColon test, stool SpecColon test showed similar sensitivity for detecting AA (53.8-61.5% vs. 58.3%) but better sensitivity (83.8-89.5% vs. 76.2%) and specificity (89.5-93.5% vs. 87.9%) for detecting CRC. Therefore, for both training and validation cohorts, stool SpecColon test demonstrated a better performance than plasma SpecColon test, probably because stool SpecColon test was performed with three PCR replicates but plasma SpecColon test with only a single PCR reaction 15. Previous studies indicated that plasma m*SEPT9* test, a FDA approved assay for CRC early detection, could achieve better performance with three PCR replicates when compared with single PCR reaction 20, 21. Meanwhile, a tissue and plasma comparison study for m*SEPT9* test showed that it could be detected in 100% of adenomas and 97.1% of CRC tissues, but it was positive in only 30.8% of adenomas and 88.2% of CRC in plasma 22, indicating a higher level of DNA methylation in tissues than that in plasma. Consistently, as stool DNA originated directly from AA and CRC tissues, the m*SFRP2* and m*SDC2* levels in stool were higher than that in plasma, thus it is reasonable that stool SpecColon test showed higher sensitivity. Nevertheless, both plasma and stool SpecColon tests showed better performance than m*SEPT9* test, especially for AA and early stage CRC detection 15, 23.

China has a large population of over 1.4 billion, and approximately 700 million people (age 45-80) should access CRC screening. However, only less than 10% eligible people in China is screened for CRC 24. The possible factors affecting the participation rate for CRC screening in China include limited medical resources, lack of time, fear of colonoscopy and financial issues 24. Fortunately, stool and plasma DNA methylation tests provide accurate, cost-effective and noninvasive methods for CRC screening. The advantage of stool DNA test is that samples can be collected at home and sent to laboratories for analysis, a convenience desirable for people who are concerned with privacy, fear of colonoscopy or lack of time. While the plasma DNA test may be a better choice for hospitals and other medical institutions, as blood draw is more convenient for medical personnel to perform. Therefore, a combination using plasma and stool DNA test based on different application scenarios by providing flexible choice for each subject may improve participation and compliance rates greatly for CRC early detection.

In 2014, a stool DNA test, Cologuard, was approved by FDA for CRC screening, which also detected two methylated DNA markers (*BMP3* and* NDRG4*), and combined with seven *KRAS* mutation sites and an immunochemical assay for human hemoglobin 25. It detected CRC and AA with sensitivities of 92.3% and 42.4%, respectively, and a specificity of 86.7% 25, and therefore it has been recommended by ACS as an option for CRC screening 3. However, methylated* BMP3*, methylated* NDRG4* and seven *KRAS* mutation sites in Cologuard test were detected by using several singleplex PCR reactions, which was much costlier (over $600) and more complicated with lower throughput than stool SpecColon test (less than $100). Meanwhile, the whole operating time for stool SpecColon test was less than 5 hours (Figure 1), which was less than 1/10 of the operating time for Cologuard test, thus it would greatly reduce time costs and increase throughput. Sun et al. recently reported a panel of stool-based DNA biomarkers for CRC screening in a format similar to Cologuard test, including the same two methylation biomarkers in our study, m*SFRP2* and m*SDC2*, seven mutation sites of *KRAS* gene and one immunochemical assay for human hemoglobin, which showed sensitivities of 91.4% and 60% respectively for CRC and AA with a specificity of 86.1% 26. In the same study, both m*SFRP2* alone and m*SDC2* alone showed the same sensitivity of 68.6% for CRC detection 26, lower than the performance of m*SFRP2* alone (71.2%-77.6%) and m*SDC2* alone (74.8%-86.2%) in this study. Meanwhile, m*SFRP2*, m*SDC2* and seven *KRAS* mutation sites in Sun et al.'s study were examined by several singleplex PCR reactions. Therefore, in developing countries with large populations and underdeveloped medical infrastructures, low-cost methods with simpler procedures such as stool SpecColon test will be more suitable for CRC early detection.

Meanwhile, in 2020, the outbreak of novel coronavirus (COVID-19) has become a Public Health Emergency of International Concern 27. Therefore, some at-home early detection methods with no physical contact, to prevent cross-infection between multiple people became important in every country. While for the stool DNA test, especially for the rapid and low cost strategy, such as stool SpecColon test, it could be sampled at home and shipped by express, which is more suitable during COVID-19 epidemic.

## Conclusion

In conclusion, this study verified the feasibility of stool SpecColon test as a novel, low cost and convenient CRC early detection strategy with high sensitivity and specificity.

## Figures and Tables

**Table 1:** Characteristics of subjects in the training cohort and the positive detection rates of m*SFRP2*, m*SDC2* and SpecColon test by different cut-off values

Group	Number (n)	Age (years)	Gender (%)			Positive detection rate (%)					
		Mean (Min-Max)	Male	Female	Cut-off-1				Cut-off-2		
					m*SFRP2*		m*SDC2*	SpecColon	m*SFRP2*	m*SDC2*	SpecColon
**Normal**	123	46.9 (23-83)	53.7	46.3	13.0		1.6	13.0	5.7	1.6	6.5
**CRC**	111	60.8 (27-84)	56.8	43.2	84.7		74.8	89.2	71.2	74.8	83.8
0	3	52.0 (38-67)	0.0	100.0	66.7		66.7	100.0	66.7	66.7	100.0
I	20	62.9 (43-79)	44.5	55.5	85.0		75.0	95.0	70.0	75.0	90.0
II	32	61.0 (35-82)	68.7	31.3	87.5		81.3	87.5	84.4	81.3	87.5
III	37	60.7 (27-83)	56.8	43.2	81.1		75.7	86.5	64.9	75.7	83.8
IV	10	58.9 (41-75)	40.0	60.0	90.0		60.0	90.0	50.0	60.0	60.0
Unknown	9	60.6 (43-84)	44.4	55.6	88.9		66.7	88.9	77.8	66.7	77.8
**Polyp**											
AA	13	58.8 (46-75)	69.2	30.8	58.8		46.2	61.5	30.8	46.2	53.8
SP	44	55.5 (24-75)	68.2	31.8	38.6		13.6	40.9	31.8	13.6	34.1
											

**Table 2:** The sensitivities, specificities and Youden indices of m*SFRP2*, m*SDC2* and SpecColon test for detecting AA and CRC by different cut-off values in the training cohort

		m*SFRP2* (%)	m*SDC2* (%)	SpecColon (%)
Cut-off-1	Sensitivity for AA (95% CI)	53.8 (26.1-79.6)	46.2 (20.4-73.9)	61.5 (32.3-84.9)
	Sensitivity for CRC (95% CI)	84.7 (76.3-90.6)	74.8 (65.5-82.3)	89.2 (81.5-94.0)
	Specificity (95% CI)	87.0 (79.4 - 92.2)	98.4 (93.7-99.7)	87.0 (79.4-92.2)
	Youden index for AA	40.8	44.6	48.5
	Youden index for CRC	71.7	73.2	76.2
Cut-off-2	Sensitivity for AA (95% CI)	30.7 (10.3-61.1)	46.2 (20.4-73.9)	53.8 (26.1-79.6)
	Sensitivity for CRC (95% CI)	71.2 (61.7-79.2)	74.8 (65.5-82.3)	83.8 (75.3-89.9)
	Specificity (95% CI)	94.3 (88.2-97.5)	98.4 (93.7-99.7)	93.5 (87.2-96.9)
	Youden index for AA	25.0	44.6	51.8
	Youden index for CRC	66.4	73.2	76.8

**Table 3:** Results of m*SFRP2*, m*SDC2* and SpecColon test in detecting CRC among different ages, genders, stage, tumor locations, tumor sides and tumor sizes by cut-off-2 in the training cohort

	m*SFRP2* (%)	*p*-value	m*SDC2* (%)	*p*-value	SpecColon (%)	*p*-value
**Age**						
<60 (n=52)	65.4 (34/52)	0.206	76.9 (40/52)	0.625	82.7 (43/52)	0.770
≥60 (n=59)	76.3 (45/59)		72.9 (43/59)		84.8 (50/59)	
**Gender**						
Male (n=63)	77.8 (49/63)	0.078	82.5 (52/63)	0.031	87.3 (55/63)	0.249
Female (n=48)	62.5 (30/48)		64.6 (31/48)		79.2 (38/48)	
**Stage**						
0-II (n=55)	78.2 (43/55)	0.128	78.2 (43/55)	0.494	89.1 (49/55)	0.151
III-IV (n=47)	61.7 (29/47)		72.3 (34/47)		78.7 (37/47)	
**Location**						
Proximal (n=56)	78.6 (44/56)	0.052	73.2 (41/56)	0.788	83.9 (47/56)	0.755
Distal (n=49)	61.2 (30/49)		75.5 (37/49)		81.6 (40/49)	
N/A (n=6)	83.3 (5/6)		83.3 (5/6)		100.0 (6/6)	
**Side**						
Left (n=86)	76.6 (66/86)	0.001	77.9 (67/86)	0.178	86.1 (74/86)	0.065
Right (n=19)	42.1 (8/19)		63.2 (12/19)		68.4 (13/19)	
N/A (n=7)	85.7 (6/7)		71.4 (5/7)		100.0 (7/7)	
**Size**						
<3 cm (n=17)	70.6 (12/17)	0.710^a^	58.8 (10/17)	0.037^a^	82.4 (14/17)	0.640^a^
3-6 cm (n=68)	75.0 (51/68)	0.700^b^	82.4 (56/68)	0.203^b^	86.8 (59/68)	0.527^b^
>6 cm (n=11)	63.6 (7/11)	0.429^c^	81.8 (9/11)	0.966^c^	90.9 (10/11)	0.701^c^
N/A (n=15)	60.0 (9/15)		53.3 (8/15)		66.7 (10/15)	

N/A, not applicable. a, *p*-value between <3 cm and 3-6 cm; b,* p*-value between <3 cm and >6 cm; c, *p*-value between 3-6 cm and >6 cm.

**Table 4:** The sensitivities and specificities of m*SFRP2* alone, m*SDC2* alone and SpecColon test for detecting SP, AA and CRC in the validation cohort

	Number (n)	Age (years)	Gender (%)		Sensitivities (%)		
		Mean (Min-Max)	Male	Female	m*SFRP2*	m*SDC2*	SpecColon
**CRC**	58	61.0 (20-79)	62.1	37.9	77.6	86.2	89.7
0	2	63.5 (57-70)	50.0	50.0	50.0	50.0	50.0
I	9	71.7 (57-79)	55.6	44.4	88.9	77.8	88.9
II	15	63.1 (36-79)	80.0	20.0	80.0	93.3	93.3
III	18	55.1 (20-72)	55.6	44.4	77.8	77.8	83.3
IV	5	60.4 (50-78)	100.0	0.0	80.0	100.0	100.0
Unknown	9	58.7 (22-78)	33.3	66.7	66.7	100.0	100.0
**Polyp**							
AA	13	66.9 (48-92)	69.2	30.8	61.5	46.2	61.5
SP	20	55.8 (31-77)	50.0	50.0	15.0	15.0	20.0
Specificities (%)	38	44.4 (22-77)	55.3	44.7	89.5	97.4	89.5

## References

[1] Siegel RL, Miller KD, Jemal A (2019). Cancer statistics, 2019. CA: a cancer journal for clinicians.

[2] Bray F, Ferlay J, Soerjomataram I, Siegel RL, Torre LA, Jemal A (2018). Global cancer statistics 2018: GLOBOCAN estimates of incidence and mortality worldwide for 36 cancers in 185 countries. CA: a cancer journal for clinicians.

[3] Wolf AM, Fontham ET, Church TR, Flowers CR, Guerra CE, LaMonte SJ (2018). Colorectal cancer screening for average-risk adults: 2018 guideline update from the American Cancer Society. CA: a cancer journal for clinicians.

[4] Cross AJ, Robbins EC, Pack K, Stenson I, Kirby PL, Patel B (2020). Long-term colorectal cancer incidence after adenoma removal and the effects of surveillance on incidence: a multicentre, retrospective, cohort study. Gut.

[5] Chen H, Li N, Ren J, Feng X, Lyu Z, Wei L (2019). Participation and yield of a population-based colorectal cancer screening programme in China. Gut.

[6] Chen W, Sun K, Zheng R, Zeng H, Zhang S, Xia C (2018). Cancer incidence and mortality in China, 2014. Chinese journal of cancer research.

[7] Qian C-N (2017). At-home cancer screening: a solution for China and other developing countries with a large population and limited number of healthcare practitioners. Chinese Journal of Cancer.

[8] Klutstein M, Nejman D, Greenfield R, Cedar H (2016). DNA methylation in cancer and aging. Cancer research.

[9] Zhang X, Song Y-F, Lu H-N, Wang D-P, Zhang X-S, Huang S-L (2015). Combined detection of plasma GATA5 and SFRP2 methylation is a valid noninvasive biomarker for colorectal cancer and adenomas. World journal of gastroenterology.

[10] Barták BK, Kalmár A, Péterfia B, Patai ÁV, Galamb O, Valcz G (2017). Colorectal adenoma and cancer detection based on altered methylation pattern of SFRP1, SFRP2, SDC2, and PRIMA1 in plasma samples. Epigenetics.

[11] Sui C, Ma J, Chen Q, Yang Y (2015). The variation trends of SFRP2 methylation of tissue, feces, and blood detection in colorectal cancer development. European Journal of Cancer Prevention the Official Journal of the European Cancer Prevention Organisation.

[12] Zhao G, Li H, Yang Z, Wang Z, Xu M, Xiong S (2019). Multiplex methylated DNA testing in plasma with high sensitivity and specificity for colorectal cancer screening. Cancer Medicine.

[13] Li H, Wang Z, Zhao G, Ma Y, Chen Y, Xue Q (2019). Performance of a MethyLight assay for methylated SFRP2 DNA detection in colorectal cancer tissue and serum. The International journal of biological markers.

[14] Chen Y, Wang Z, Zhao G, Sun C, Ma Y, Zhang L (2019). Performance of a Novel Blood-Based Early Colorectal Cancer Screening Assay in Remaining Serum after the Blood Biochemical Test. Disease markers.

[15] Zhao G, Ma Y, Li H, Li S, Zhu Y, Liu X (2020). A novel plasma based early colorectal cancer screening assay base on methylated SDC2 and SFRP2. Clinica Chimica Acta.

[16] Potter NT, Hurban P, White MN, Whitlock KD, Loftonday CE, Tetzner R (2014). Validation of a Real-Time PCR-Based Qualitative Assay for the Detection of Methylated SEPT9 DNA in Human Plasma. Clinical chemistry.

[17] TR C, M W, C L-D, SJ M, M B SR P (2014). Prospective evaluation of methylated SEPT9 in plasma for detection of asymptomatic colorectal cancer. Gut.

[18] Zhao G, Liu X, Liu Y, Li H, Zheng M (2020). Aberrant DNA Methylation of SEPT9 and SDC2 in Stool Specimens as an Integrated Biomarker for Colorectal Cancer Early Detection. Frontiers in Genetics.

[19] Oh TJ, Oh HI, Yang YS, Jeong D, Kim C, Kang HW (2017). Feasibility of quantifying SDC2 methylation in stool DNA for early detection of colorectal cancer. Clinical Epigenetics.

[20] Song L, Li Y (2017). Progress on the clinical application of the SEPT9 gene methylation assay in the past 5 years. Biomarkers in Medicine.

[21] Song L, Yu H, Jia J, Li Y (2017). A systematic review of the performance of the SEPT9 gene methylation assay in colorectal cancer screening, monitoring, diagnosis and prognosis. Cancer Biomarkers.

[22] Tóth K, Wasserkort R, Sipos F, Kalmár A, Wichmann B, Leiszter K (2014). Detection of methylated septin 9 in tissue and plasma of colorectal patients with neoplasia and the relationship to the amount of circulating cell-free DNA. PloS one.

[23] Potter NT, Hurban P, White MN, Whitlock KD, Lofton-Day CE, Tetzner R (2014). Validation of a real-time PCR-based qualitative assay for the detection of methylated SEPT9 DNA in human plasma. Clinical chemistry.

[24] Sano Y, Byeon JS, Li XB, Wong MC, Chiu HM, Rerknimitr R (2016). Colorectal cancer screening of the general population in East Asia. Digestive Endoscopy.

[25] Imperiale TF, Ransohoff DF, Itzkowitz SH, Levin TR, Lavin P, Lidgard GP (2014). Multitarget stool DNA testing for colorectal-cancer screening. New England Journal of Medicine.

[26] Sun M, Liu J, Hu H, Guo P, Shan Z, Yang H (2019). A novel panel of stool-based DNA biomarkers for early screening of colorectal neoplasms in a Chinese population. Journal of cancer research and clinical oncology.

[27] Lancet T (2020). Emerging understandings of 2019-nCoV. Lancet (London, England).

